# Double closed loop PI control with full compensation arc elimination based on zero sequence voltage of neutral point

**DOI:** 10.1038/s41598-024-66186-8

**Published:** 2024-07-12

**Authors:** Hongwen Liu, Xiangjun Zeng, Qing yang, Chenchao Chai

**Affiliations:** 1 State Key Laboratory of Power Transmission Equipment and System Security and New Technology, Chongqing University, Chongqing, 400044 China; 2 Electric Power Research Insitute, Yunnan Power Grid Co., Ltd, Kunming, 650217 China; 3 School of Electrical and Information Engineering, Changsha University of Science and Technology, Changsha, 410114 China; 4 Yunnan Megasun Technology Co., Ltd., Kunming, 650217 Yunan Province China

**Keywords:** Unbalanced distribution network, Voltage deviation, Full compensation arc suppression, Double closed loop PI control, Electrical and electronic engineering, Power distribution

## Abstract

Aiming at the problem of zero sequence voltage generated by unbalance parameters of line to ground, which affects arc suppression effect of grounding fault of controllable voltage source. By analyzing the influence of ground unbalance parameters on the arc suppression effect of controllable voltage source under different grounding modes, the mechanism of full compensation arc suppression based on zero sequence voltage of neutral point is revealed, and on this basis, a fully compensated arc suppression model of controllable voltage source controlled by double closed loop PI is established, and the deviation control is carried out by using the neutral voltage of distribution network and the voltage of fault phase supply. The residual voltage ring adopts the ground fault phase residual voltage for closed loop control. The simulation results show that the dual-closed-loop PI control algorithm can continuously stabilize the output waveform of the controllable voltage source. When the transition resistance is 0.1 ~ 10 kΩ, the residual voltage stabilization time of the independent controllable voltage source grounding method is 43 ms ~ 2.4 s, and the parallel arc suppression coil grounding method is 43 ms ~ 4.7 s. The proposed dual closed-loop PI control method for neutral point voltage deviation and fault residual voltage can stabilize the residual voltage of the grounded fault phase to below 10 V, forcing reliable arc extinction at the grounded fault point, exhibiting good stability. Low-voltage simulation tests have also proved the feasibility of the algorithm.

## Introduction

Because the ground fault can last for several hours, the arc current at the fault point may cause an interphase fault, leading to a power outage, which affects the reliability of the power supply^1–3^. In addition, the arc around the fault point may even cause forest fires and human electrocution accidents^4,5^. Therefore, it is of great practical significance to study the full arc elimination technology of grounding fault arc current in distribution network to ensure the reliability of power supply^6–8^.

Distribution network grounding fault arc suppression method can be divided into active arc suppression method and passive arc suppression method according to the compensation device^9,10^. In^11,12^,expounds the active arc suppression mechanism based on the compensation current injection of three-phase active arc suppression device and proposes a new active arc suppression method with the co-operation of the arc suppression coil and the three-phase active arc suppression device. In^13^, an active compensation device is used to inject zero sequence current into the neutral point of the distribution network to compensate the fault current, but this method needs to measure the ground parameters of the distribution network and has certain limitations. In^14^ proposed a compensation current based on the measurement deviation of conductance and susceptance before and after faults, which can reduce the dependence on the measurement accuracy of parameters. In^15^ proposed an active arc suppression method for grounding faults of distribution network based on secondary injection, which only needs to inject two power frequency currents to the neutral point before the fault to complete the measurement of capacitance current to the ground. There is no need to measure the distribution network to the ground parameters, and the method is simple. In^16–18^ proposes a continuously adjustable impedance earthing arc suppression device, which can realize full compensation of earthing fault capacitive current by adjusting the impedance of arc suppression coil or magnetron reactor.

At the same time, in order to solve the problems of low compensation accuracy of arc suppression coil and large residual current under complex working conditions, a variety of active full compensation methods based on modern power electronics technology have been proposed^19,20^. Among them, the most representative is a ground fault neutralizer (GFN). The ground fault residual current in this method cannot be directly obtained, and the residual current value needs to be calculated based on the distribution parameters of the system to the ground, with a large deviation^21,22^. Swedish Neutra Company developed a ground fault neutralizer (GFN) based on the theory of fault residual current compensation, and compensated the capacitive reactive current and active component residual current in the system through the combination of arc elimination coil and residual current compensator^23^. In^24^, To improve the self-healing ability of the distribution network, this paper proposes a flexible arc suppression method based on double closed-loop control and fault status identification, which directly controls the neutral point voltage of distribution network. The reignition of fault arc can be suppressed by flexible arc suppression device (FASD). In^25^, a method was proposed to compensate for the grounding fault current by connecting a reactive compensation element in the lagging phase of the fault phase. However, this solution relies on accurate measurement of system parameters, and the compensating element is a passive component with reactive impedance, for which there is currently no engineering-friendly method for continuous adjustment. In^26^, similar to GFN, an inverter is used to inject compensation current from the neutral point to compensate for the residual grounding fault current, and a nonlinear model predictive controller is introduced. However, it is still limited by the accuracy of system parameters.

It is not difficult to find from the above literature research that many scholars have conducted a lot of research on the problem that the arc suppression mode of arc suppression coil cannot achieve full compensation, and have achieved fruitful results. However, the following two problems still exist: (1) The natural unbalanced zero-sequence voltage of distribution network affects the arc suppression effect and the automatic tracking and control of fault point current of active current source is complicated; (2) In engineering practice, it is difficult to accurately measure the capacitance current of the system and track the fault phase voltage in real time due to the measurement error and the real-time change of the system state. In view of the above problems, by analyzing the influence of unbalance parameters to ground on the arc suppression effect of controllable voltage source under different grounding modes, the mechanism of full compensation arc suppression based on zero sequence voltage of neutral point is revealed, and on this basis, a fully compensated arc suppression model of controllable voltage source controlled by double closed loop PI is established. The deviation loop adopts the neutral voltage of distribution network and the fault phase supply voltage to control the deviation, and the residual voltage loop adopts the ground fault phase residual voltage to control the closed loop.

## Analysis of the influence of different grounding modes on unbalance parameters

### Independent controllable voltage source grounding mode

**Figure 1:**
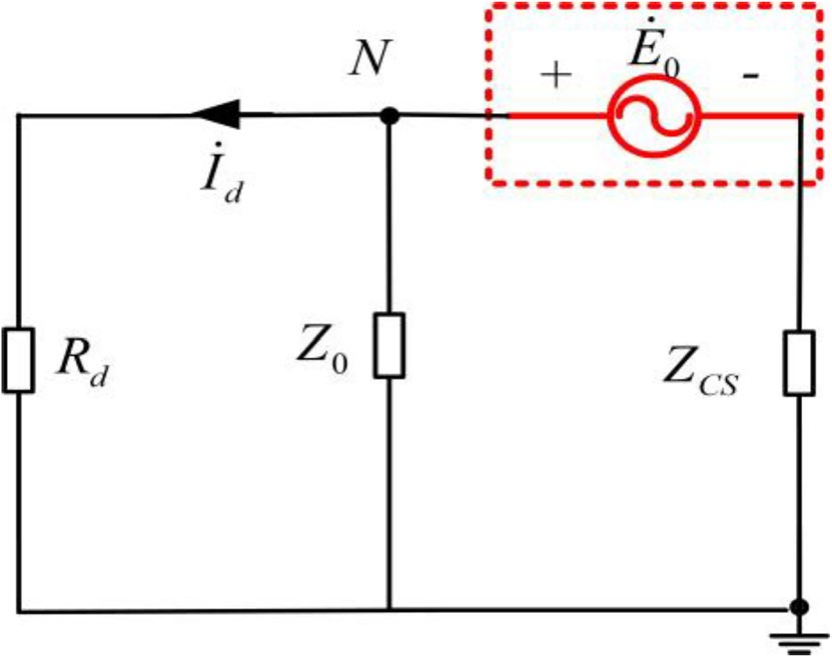
Equivalent circuit of independent controllable voltage source compensation mode.

For the independent controllable voltage source grounding mode, when the ground parameters of the distribution network line are unbalanced, the equivalent zero-sequence power supply voltage will be generated between the neutral point and the ground. After the grounding fault occurs, when the controllable voltage source fully compensates for arc elimination, an equivalent zero-sequence power supply is superimposed at the fault point, and the output voltage of the equivalent zero-sequence power supply is determined by the imbalance degree of system parameters. Usually tens of volts to thousands of volts. Let the equivalent zero-sequence power supply voltage be, and the equivalent circuit affecting the residual voltage and residual current analysis at the fault point can be shown in Fig. 1.

In Fig. 1, are the ground fault transition resistance *R*_*d*_, the internal impedance of the voltage source *Z*_0_, and the zero-sequence impedance of the system to ground *Z*_*CS*_. It can be seen that the voltage of neutral point N is the residual voltage of the fault point under the action of equivalent zero-sequence power supply voltage, then the neutral point voltage $\dot{U}_{N}$ is:

1 $$\dot{U}_{cy} = \dot{U}_{N} = \frac{{\frac{{\dot{E}_{0} }}{{Z_{CS} }}}}{{\frac{1}{{Z_{CS} }} + \frac{1}{{R_{d} }} + \frac{1}{{Z_{0} }}}}$$

The residual current at the fault point under the effect of equivalent zero sequence voltage is:

2 $$\dot{I}_{cl} = \dot{I}_{d} = \frac{{\dot{U}_{N} }}{{R_{d} }} = \frac{{\dot{E}_{0} Z_{0} }}{{Z_{CS} Z_{0} + Z_{0} R_{d} + Z_{CS} R_{d} }}$$

According to formula (2), the residual voltage at the fault point is related to the zero sequence impedance of the system to the ground, the internal impedance of the voltage source and the ground transition resistance. When the internal impedance of the controlled voltage source *Z*_0_ = 0 , the influence of the equivalent zero-sequence power supply voltage generated by the unbalanced distribution network on the residual voltage and residual current can be ignored; when the equivalent zero-sequence power supply voltage generated by the unbalanced distribution network $\dot{E}_{0}$ and the internal resistance of the controlled voltage source *Z*_0_ are determined, the residual voltage at the fault point is related to *Z*_*CS*_ and *R*_*d*_.

### Controlled voltage source parallel arc suppression coil grounding method

**Figure 2:**
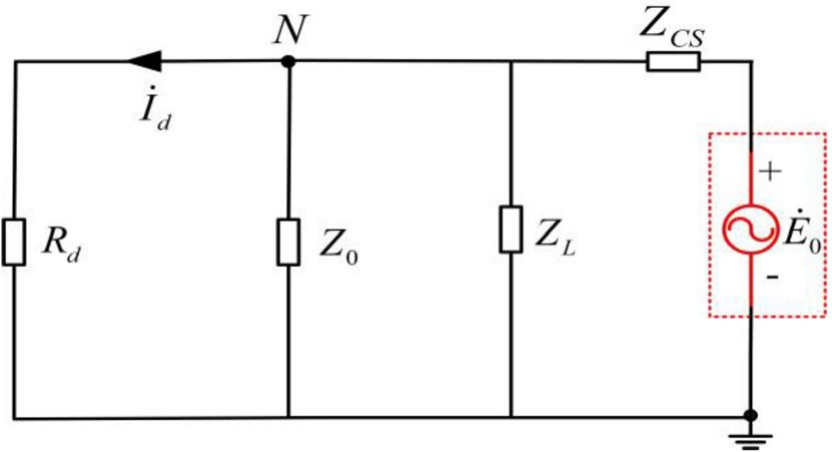
Equivalent circuit of controlled voltage source parallel arc suppression coil grounding system.

For the parallel arc suppression coil grounding system with controlled voltage sources, the equivalent circuit for the analysis of the effect of the equivalent zero sequence power supply voltage on the residual voltage and residual current at the fault point is shown in Fig. 2.

In Fig. 2, *R*_*d*_ is the ground fault transition resistance, *Z*_0_ is the internal impedance of the voltage source, *Z*_*CS*_ the system zero sequence impedance to the ground, and *Z*_*L*_ is the arc suppression coil impedance. Similarly, the voltage of neutral point *N* is the residual voltage of the fault point under the action of equivalent zero-sequence power supply voltage, and the neutral point voltage $\dot{U}_{N}$ is:

3 $$\dot{U}_{cy} = \dot{U}_{N} = \frac{{\frac{{\dot{E}_{0} }}{{Z_{CS} }}}}{{\frac{1}{{Z_{CS} }} + \frac{1}{{R_{d} }} + \frac{1}{{Z_{0} }} + \frac{1}{{Z_{L} }}}}$$

The residual current at the fault point under the effect of equivalent zero sequence voltage is:

4 $$\dot{I}_{cl} = \dot{I}_{d} = \frac{{\dot{U}_{N} }}{{R_{d} }} = \frac{{\dot{E}_{0} Z_{0} Z_{L} }}{{Z_{CS} Z_{0} R_{d} + Z_{L} Z_{0} R_{d} + Z_{CS} Z_{L} R_{d} + Z_{CS} Z_{0} Z_{L} }}$$

According to Eq. (4), the residual voltage at the fault point is related to the zero-sequence impedance of the system to the ground, the impedance of arc suppression coil, the impedance in the voltage source and the grounding transition resistance of the system, and the grounding mode is the same as that of the independent controllable voltage source. When the impedance in the controllable voltage source is Z_0_ = 0, the influence of the equivalent zero-sequence power supply voltage generated by the unbalanced distribution network on the residual voltage and residual current can be ignored. When the equivalent zero sequence power supply voltage $\dot{E}_{0}$ and the internal resistance of the controllable voltage source generated by the unbalanced distribution network *Z*_0_ are determined, the residual voltage at the fault point is related to *Z*_*CS*_, *R*_*d*_ and *Z*_*L*_.

## Arc-elimination mechanism based on zero sequence voltage of neutral point

**Figure 3:**
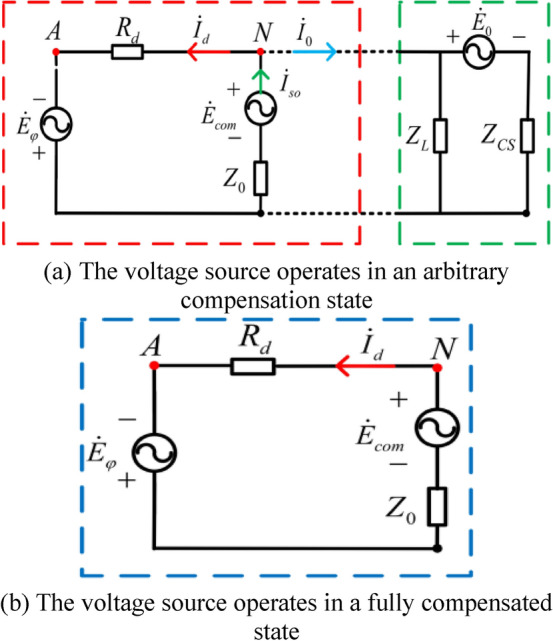
Equivalent circuit of controllable voltage source in compensation state.

To control the output of the controllable voltage source in real time according to the change of operating conditions, it is necessary to analyze the grounding fault current $\dot{I}_{d}$, the output current of the controllable voltage source $\dot{I}_{so}$ and the full compensation current $\dot{I}_{0}$. The equivalent circuit of controllable voltage source in compensation state after ground fault occurs is shown in Fig. 3.

As shown in Fig. 3a, when the neutral point voltage $\dot{U}_{N}$ is not equal to the fault phase supply voltage $- \dot{E}_{\varphi }$, the output of the controllable voltage source $\dot{E}_{com}$ is not in the fully compensated state, the ground fault current $\dot{I}_{d}$ not 0, and the arc continues to burn, then:

5 $$\dot{I}_{so} = \dot{I}_{d} + \dot{I}_{0} = \dot{I}_{d} + \frac{{\dot{U}_{N} }}{{Z_{CS} }} + \frac{{\dot{U}_{N} - \dot{E}_{0} }}{{Z_{CS} }} = \dot{I}_{d} + \frac{{\dot{E}_{0} }}{{Z_{CS} }}$$

Because $$\dot{I}_{so} = \frac{{\dot{U}_{N} - \dot{E}_{com} }}{{Z_{0} }}$$, then:

6 $$\dot{I}_{d} = \frac{{\dot{U}_{N} - \dot{E}_{com} }}{{Z_{0} }} - \frac{{\dot{E}_{0} }}{{Z_{CS} }}$$

When $\dot{I}_{d} = 0$, the output voltage when the controllable voltage source is fully compensated $\dot{E}_{{com{ - }op}}$:

7 $$\dot{E}_{{com{ - }op}} = \dot{U}_{N} - \dot{E}_{0} \frac{{Z_{0} }}{{Z_{CS} }} = \dot{E}_{\varphi } - \dot{E}_{0} \frac{{Z_{0} }}{{Z_{CS} }}$$

The controllable voltage source $\dot{E}_{com}$ can control the neutral voltage of the distribution network $\dot{U}_{N}$ through Eq. (7). If the current equation of the neutral node in column N of the distribution network is $\dot{I}_{so} = \dot{I}_{d} + \dot{I}_{0}$, if the fault current is fully compensated $\dot{I}_{d} = 0$, $\dot{I}_{so} = \dot{I}_{0}$, the equivalent AN branch in Fig. 3b is open, then the neutral voltage $\dot{U}_{N}$ = $\dot{U}_{A}$ = $- \dot{E}_{\varphi }$, and the neutral voltage is the reverse voltage of the fault-phase supply voltage, with equal amplitude and opposite phase.

**Figure 4:**
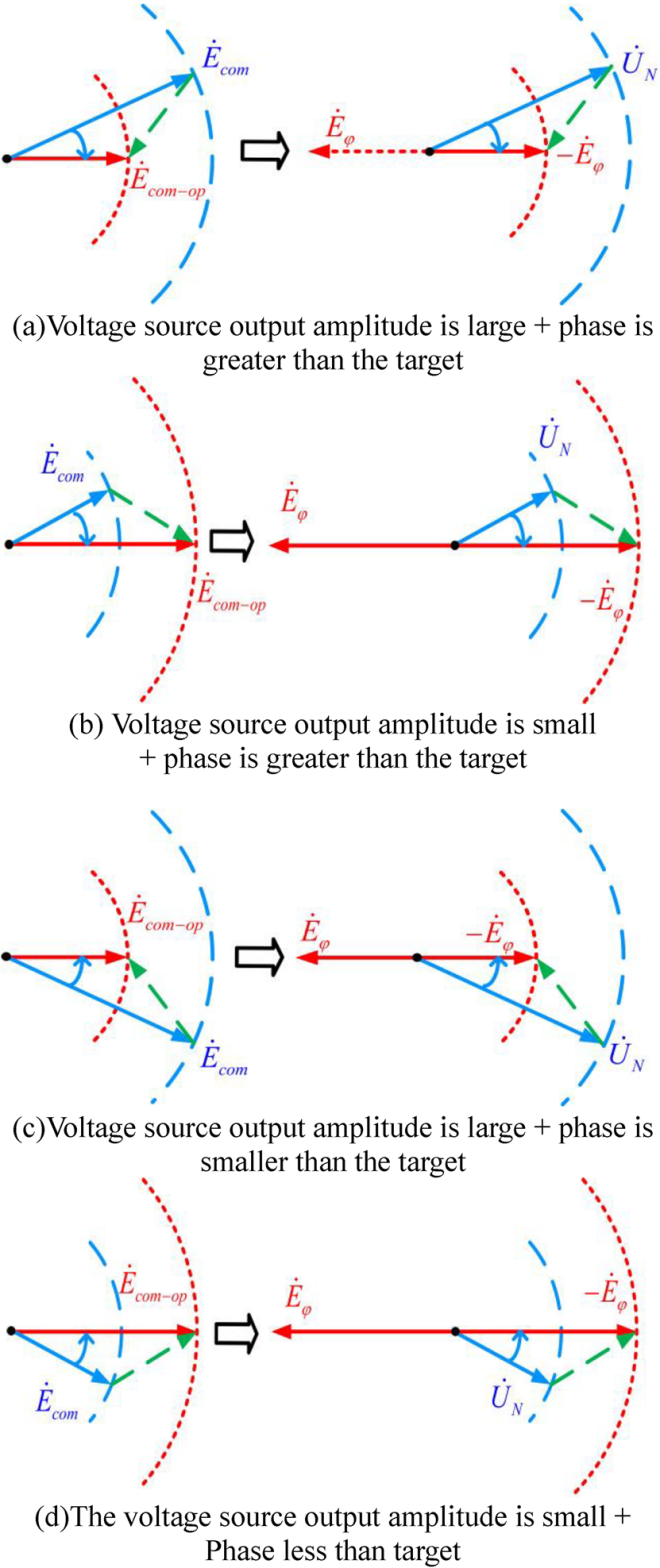
Vector relationship of neutral voltage controlled by voltage source.

In Fig. 3b When the controllable voltage source is fully compensated, the output voltage of the voltage source $\dot{E}_{{com{ - }op}}$ is assumed to be, the output voltage of the voltage source $\dot{E}_{com}$ is deviated from the target value $\dot{E}_{{com{ - }op}}$, and the internal impedance of the voltage source is usually small, $\dot{U}_{N} \approx \dot{E}_{com}$ then $\dot{E}_{{com{ - }op}} \approx - \dot{E}_{\varphi }$, The deviation between $\dot{E}_{com}$ and $\dot{E}_{{com{ - }op}}$ can be regarded as the deviation between $\dot{U}_{N}$ the $- \dot{E}_{\varphi }$ in the neutral voltage of the system..The vector relationship of $\dot{E}_{{com{ - }op}}$, $\dot{E}_{com}$, $\dot{E}_{\varphi }$, $\dot{U}_{N}$ and is shown in Fig. 4.

Figure 4a,b, when $\dot{E}_{com}$ phase is ahead of $\dot{E}_{{com{ - }op}}$ phase, then $\dot{U}_{N}$ phase is also ahead of $- \dot{E}_{\varphi }$ phase, that is $\dot{E}_{{com{ - }op}} - \angle \dot{E}_{com} < 0$, then $\angle - \dot{E}_{\varphi } - \angle \dot{U}_{N} < 0$, at this time to reduce $\dot{E}_{com}$ phase to achieve $\dot{E}_{{com{ - }op}}$ target phase.

Figure 4c,d, when $\dot{E}_{com}$ phase lags behind $\dot{E}_{{com{ - }op}}$ phase, then $\dot{U}_{N}$ phase also lags behind $- \dot{E}_{\varphi }$ phase, that is $\angle \dot{E}_{{com{ - }op}} - \angle \dot{E}_{com} > 0$, then $\angle - \dot{E}_{\varphi } - \angle \dot{U}_{N} > 0$, at this time need to increase $\dot{E}_{com}$ phase to achieve $\dot{E}_{{com{ - }op}}$ target phase.

Figure 4a,c, when $\dot{E}_{com}$ amplitude is greater than $\dot{E}_{{com{ - }op}}$ amplitude, then $\dot{U}_{N}$ amplitude is also greater than $\dot{E}_{\varphi }$ amplitude, that is $\left| {\dot{E}_{{com{ - }op}} } \right| - \left| {\dot{E}_{com} } \right| < 0$, then $\left| {\dot{E}_{\varphi } } \right| - \left| {\dot{U}_{N} } \right| < 0$. At this time, $\dot{E}_{com}$ amplitude needs to be reduced to reach $\dot{E}_{{com{ - }op}}$ target amplitude.

Figure 4b,d, when $\dot{E}_{com}$ amplitude is less than $\dot{E}_{{com{ - }op}}$ amplitude, then $\dot{U}_{N}$ amplitude is also less than $\dot{E}_{\varphi }$ amplitude, that is, $\left| {\dot{E}_{com\_op} } \right| - \left| {\dot{E}_{com} } \right| > 0$, then $\left| {\dot{E}_{\varphi } } \right| - \left| {\dot{U}_{N} } \right| > 0$. At this time, $\dot{E}_{com}$ amplitude needs to be increased to reach $\dot{E}_{{com{ - }op}}$ target amplitude.

Therefore, by calculating the deviation between $\dot{E}_{\varphi }$ amplitude and $\dot{U}_{N}$, real-time correction of the voltage source output $\dot{E}_{com}$ amplitude, while calculating the phase deviation between $- \dot{E}_{\varphi }$ and $\dot{U}_{N}$ real-time correction of the voltage source output $\dot{E}_{com}$ phase, then the voltage source output $\dot{E}_{com}$ approach the ideal value $\dot{E}_{{com{ - }op}}$, to achieve grounding fault arc elimination.

## Double closed loop PI control full complement arc elimination model

In this section, a controlled voltage source arc suppression control method using double closed loop PI control is proposed. The deviation loop adopts the deviation control of distribution network neutral voltage and fault phase supply voltage, and the residual voltage loop adopts the ground fault residual voltage to further close the loop control. The two closed-loop control objects have different characteristics, which helps to improve the performance of the system**.**

### Double closed loop PI control system block diagram

**Figure 5:**
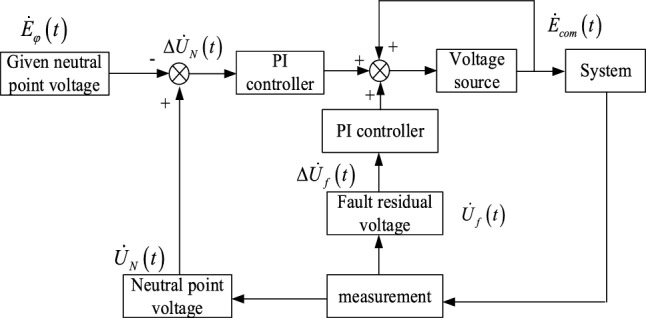
Block diagram of controlled voltage source full compensation arc suppression control system.

The system is mainly composed of neutral voltage control part, ground fault residual voltage control part, voltage source initial value part and measurement part. Both the amplitude and phase control of the output voltage of the voltage source are double closed-loop control, constituting a double closed-loop control system, as shown in Fig. 5.

### Double closed loop PI full compensation arc suppression control model

Since the parameters of the output voltage mathematical model of the voltage source of the controlled object change with time, PI control method is suitable for use. The form of PI control loop algorithm based on neutral point voltage deviation is as follows:

8 $$\dot{E}_{com} (t) = \dot{E}_{com} (t - 1) + K_{P} \Delta \dot{U}_{N} (t) + K_{I} \frac{1}{T}\int\limits_{0}^{t} {\Delta \dot{U}_{f} (t)dt}$$

In formula (8), $\dot{E}_{com} (t)$ is the output control of the current voltage source; $\dot{E}_{com} (t - 1)$ is the output voltage of the voltage source at the previous time; $\Delta \dot{U}_{N} (t)$ Is the error between the measured value of the neutral voltage and the ideal output voltage of the set value $\dot{E}_{{com{ - }op}}$;$\frac{1}{T}\int_{0}^{t} {\Delta \dot{U}_{N} (t)dt}$ is the arithmetic average sum of voltage errors at all neutral points in the time period T.

Ground fault residual pressure PI control ring algorithm type is:

9 $$\dot{U}_{f} (t) = K_{P} \Delta \dot{U}_{f} (t) + K_{I} \frac{1}{T}\int\limits_{0}^{t} {\Delta \dot{U}_{f} (t)dt}$$

In formula (9), $\dot{U}_{f} (t)$ is the current residual voltage output control quantity, $\Delta \dot{U}_{f} (t)$ is the error between the measured value of ground fault residual pressure and the set expected residual pressure value. $\frac{1}{T}\int_{0}^{t} {\Delta \dot{U}_{f} (t)dt}$ is the arithmetic average sum of all residual pressure errors over the time period *T*.

**Figure 6:**
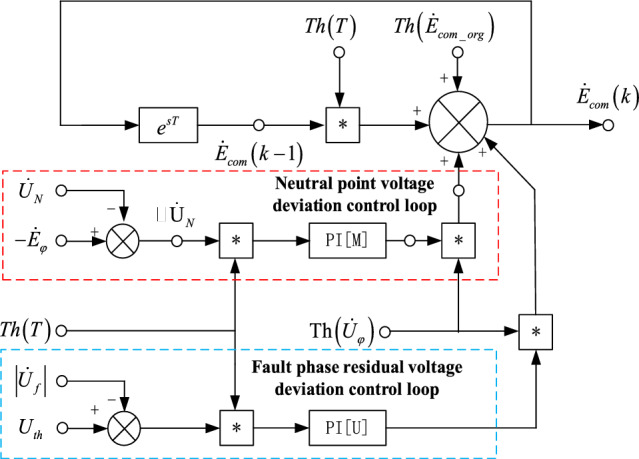
Block diagram based on double closed loop PI control.

*K*_*P*_ and *K*_*I*_ are the PI controller parameter, *K*_*P*_ proportional term reflects the response rate of voltage regulation of the output voltage of the voltage source, *K*_*I*_ the integral term eliminates the steady-state error of the output voltage of the voltage source. The output voltage double closed-loop control block diagram of controllable voltage source is shown in Fig. 6.

In Fig. 6, the initial output voltage $\dot{E}_{com - op}$ of the voltage source is calculated according to Eq. (7), measure the neutral voltage $\dot{U}_{N}$ and the fault phase supply voltage $\dot{E}_{\varphi }$, calculate the deviation between the neutral voltage and the fault phase supply voltage $\Delta \dot{U}_{N} = \Delta \sigma \angle \varphi$. The initial voltage source assignment function $Th(\dot{E}_{com - op} )$ is:

10 $$Th(\dot{E}_{com - op} ) = \left\{ {\begin{array}{*{20}l} {\dot{E}_{com - op} ,} \hfill & {t < 0.4\;s} \hfill \\ {0,} \hfill & { t \ge 0.4\;s} \hfill \\ \end{array} } \right.$$

$Th(T)$ for the voltage source to start automatic regulation after starting compensation, the governing equation is:

11 $$Th(T) = \left\{ {\begin{array}{*{20}l} {0,} \hfill & {t < 0.4\;s} \hfill \\ {1,} \hfill & { t \ge 0.4\;s} \hfill \\ \end{array} } \right.$$

$\left| {\dot{U}_{f} } \right|$ is the fault phase voltage amplitude; $U_{th}$ is the preset fault residual pressure threshold, usually setting to 10 V; $Th(\dot{U}_{\varphi } )$ to stop regulating the voltage source when the fault phase is lower than the threshold, the mathematical expression is:

12 $$Th(\dot{U}_{\varphi } ) = \left\{ \begin{gathered} \, 0, \left| {\dot{U}_{f} } \right| \le U_{th} \hfill \\ 1, \left| {\dot{U}_{f} } \right| > U_{th} \hfill \\ \end{gathered} \right.$$

If the fault phase voltage is greater than the threshold $U_{th}$, adjust the voltage source. When the fault phase voltage is lower than the threshold, stop regulating the voltage source.

The error $\Delta \dot{U}_{N}$ between the neutral voltage and the fault phase supply voltage is multiplied by the PI[M] controller to obtain $Th(\dot{U}_{\varphi } )$ the voltage source voltage correction value one.

The fault residual voltage threshold $U_{th}$ and fault residual voltage $\left| {\dot{U}_{f} } \right|$ error are multiplied by PI[U] controller to obtain $Th(\dot{U}_{\varphi } )$ the voltage source voltage correction value two.

**Figure 7:**
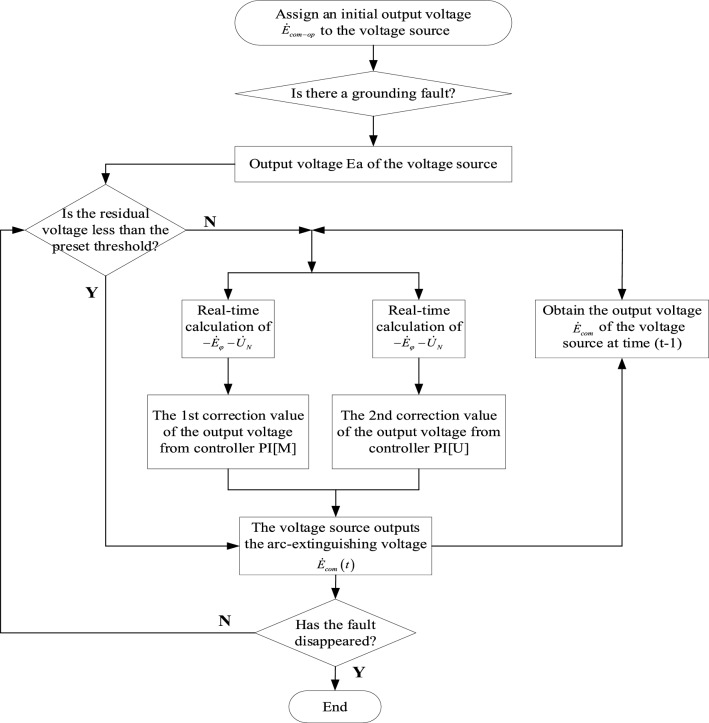
Double closed loop PI full compensation arc elimination control process.

$\dot{E}_{com} (t)$ is the output voltage of the voltage source at time t, and the output voltage of the voltage source $\dot{E}_{com} (t - 1)$ at time t is obtained after the e-sTdelay link, and the target voltage of the voltage source at time t is obtained after the superposition of the correction values of the voltage deviation ring and fault residual voltage ring at time* t*. The algorithm flow of the controllable voltage source double closed-loop PI fully compensated arc suppression control system is shown in Fig. 7.

## Simulation analysis

### Basis of simulation

**Figure 8:**
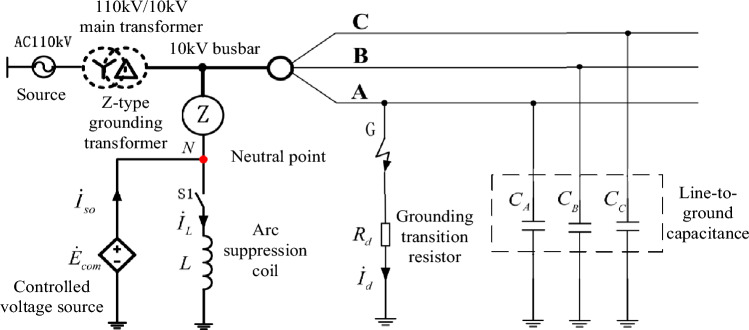
Simulation model body.

In this section, PSCAD/EMTP is used to establish a double closed-loop PI control automatic tracking fully compensated arc suppression model based on neutral point voltage deviation, and to simulate and analyze the fully compensated arc suppression under the independent controlled voltage source grounding mode and the parallel controlled voltage source arc suppression coil grounding mode of unbalanced distribution network. The simulation model is shown in Fig. 8. In the figure, the system power supply is 110 kV three-phase symmetrical power supply, the main transformer ratio is 110 kV/10 kV, and the capacity is 50 MVA. The neutral point N of the 10 kV distribution network is drawn out through the ground transformer, and the controllable voltage source $\dot{E}_{com}$ is connected to the neutral point *N* of the system and the earth. Arc suppression coil *L* is connected to the system neutral point by switch S1.

The zero sequence impedance of the distribution network to the ground is concentrated capacitance, and the distribution network with the metallic fault current of 98 A is simulated. Four working conditions with different imbalance degrees are analyzed respectively. Working condition 1: the total ground capacitance of the simulated balanced distribution network is 54 uF. Working condition 2: the unbalance degree of three relative parameters of simulated unbalanced distribution network is 3%; Working condition 3: the unbalance degree of the three relative ground parameters of the simulated unbalanced distribution network is 6%; Working condition 4: the unbalance degree of three relative parameters of simulated unbalanced distribution network is 9%; Represents the ratio of the unbalance voltage amplitude to the rated phase voltage of the system. When theTable 1o Simulation model parametersource is in parallel with the arc suppression coil, the inductance of the arc suppression coil is selected as 0.176H, in the overcompensation state, and the detuning degree is − 6.5%. Parameters of the simulation model are shown in Table 1.

**Table 1:** Simulation model parameters.

Parameter	Value			
	Working condition 1	Working condition 2	Working condition 3	Working condition 4
C/uF	C_A_ = 18uF C_B_ = 18uF C_C_ = 18uF	C_A_ = 17.1uF C_B_ = 18uF C_C_ = 18.9uF	C_A_ = 16.2uF C_B_ = 18uF C_C_ = 19.8uF	C_A_ = 15.3uF C_B_ = 18uF C_C_ = 20.7uF
$\varepsilon_{u}$*/*%	0%	3%	6%	9%
$\dot{E}_{0}$*/V*	0	167∠149°V	333∠149°V	499∠149°V
*L*/H	0.176H			
*R*_d_/Ω	100Ω, 500Ω, 1kΩ, 5kΩ, 10kΩ			

### Analysis of influence of unbalance parameters on arc elimination effect

If the metallic ground fault occurs in the independent controllable voltage source grounding mode of unbalanced distribution network, the fault point is the capacitive current of about 106 A, and the fault point current decreases with the increase of the grounding transition resistance. In order to comprehensively analyze the influence of distribution network on the unbalance of ground parameters on the arc suppression effect under the grounding mode of independent controllable voltage source, the fault phase residual voltage of controllable voltage source under fully compensated arc suppression under working conditions 1–4 is analyzed when the grounding transition resistance is 100 Ω, 500 Ω, 1 kΩ, 5 kΩ and 10 kΩ respectively.The equivalent zero-sequence power supply voltage of distribution network $\dot{E}_{0}$ and the initial output fully compensated arc suppression voltage of controllable voltage source $\dot{E}_{com - op}$ are shown in Table 2.

**Table 2:** The initial voltage under various working conditions under different grounding modes.

	Working condition 1	Working condition 2	Working condition 3	Working condition 4
$\dot{E}_{0}$/V	0	167∠149°	333∠149°	499∠149°
Grounding mode 1	4.236∠180°	4.275∠179.7°	4.313∠179.4°	4.345∠179.1°
Grounding mode 2	5.874∠180°	5.912∠179.8°	5.95∠179.6°	5.982 ∠179.4°

**Figure 9:**
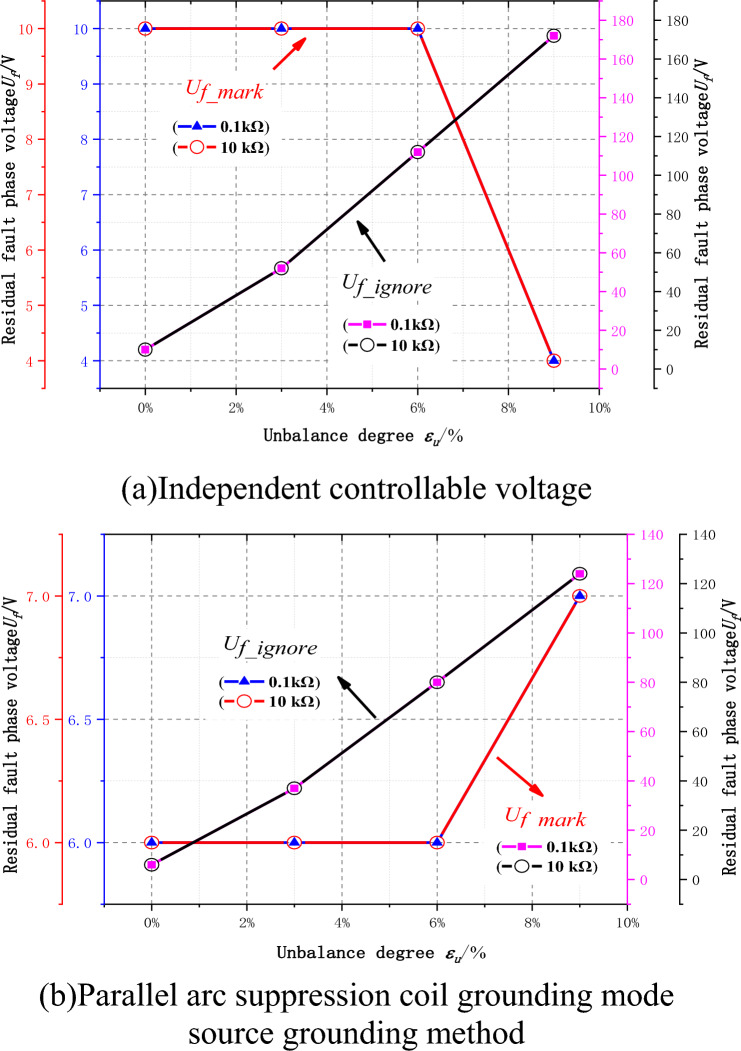
Relationship between unbalance degree and fault residual pressure.

The following is a simulation of the influence of unbalance degree $\varepsilon_{u}$ of different ground parameters on the fully compensated arcing effect of the controllable voltage source in the distribution network under the independent controllable voltage source grounding mode and the parallel arc suppression coil grounding mode. Figure 9 shows the fault phase residual voltage $\dot{U}_{f}$ of the controllable voltage source fully compensated under the two grounding modes with each unbalance degree ranging from 0 to 9%.

It can be seen from Fig. 9a that the fault residual voltage $\dot{U}_{f\_ignore}$ without considering the influence of unbalance is up to 172 V. With the increase of unbalance, the fault residual voltage increases, and the grounding transition resistance has nothing to do with the residual voltage. The simulation results show that the fully compensated arc suppression method of the controllable voltage $\dot{U}_{f\_mark}$ source proposed in this paper is less than 10 V under all stable conditions, which is equivalent to the residual voltage of the balanced distribution network under condition 1, and verifies the correctness of the fully compensated arc suppression calculation method under the neutral voltage source grounding mode of the unbalanced distribution network with ground parameters.

It can be seen from Fig. 9b that the fault residual voltage $\dot{U}_{f\_ignore}$ without considering the influence of unbalance is up to 124 V, which is lower by 48 V compared with the fully compensated independent controllable voltage source. With the increase of unbalance, the fault residual voltage increases, and the grounding transition resistance has nothing to do with the residual voltage. The simulation calculation shows that by using the PI control method of controlled voltage source output based on neutral voltage deviation proposed in this paper, the fault residual voltage $\dot{U}_{f\_mark}$ under stable conditions is less than 10 V, which is equivalent to the residual voltage of balanced distribution network under condition 1. The correctness of the calculation method of full compensation arc suppression under the parallel arc suppression coil grounding of neutral controllable voltage source in unbalanced distribution network with ground parameters is verified.

### Analysis of arc elimination effect

In order to further analyze the response characteristics of PI controller based on neutral voltage deviation under neutral independent controllable voltage source grounding. Assuming A ground fault occurs in phase A, the PI controller's output amplitude and phase stability performance are calculated when the grounding transition resistance is 0.1–10 kΩ and the maximum unbalance $\varepsilon_{u}$ is 9%.

**Figure 10:**
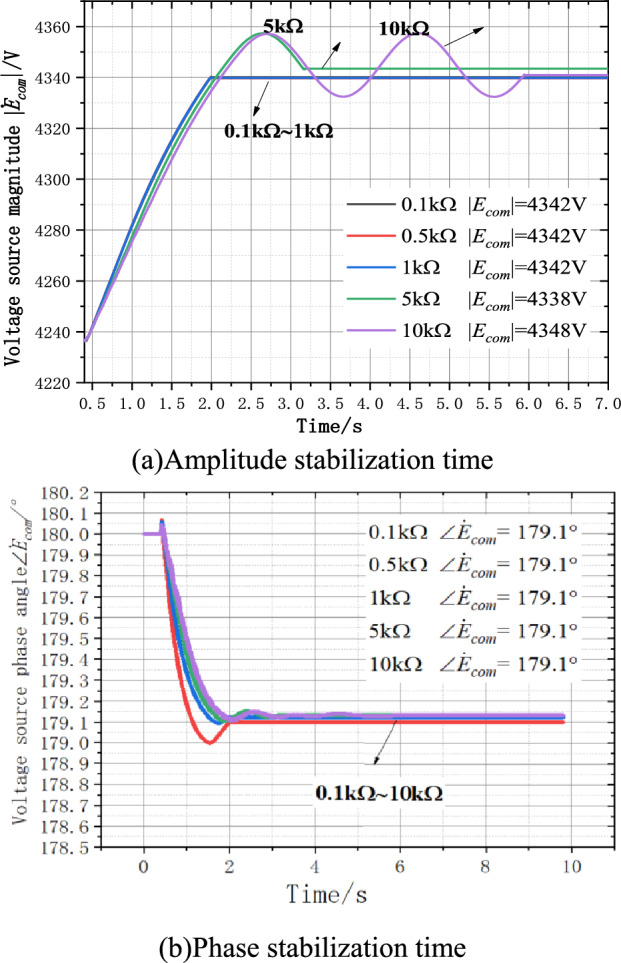
PI controller output and time relationship.

Under the independent controllable voltage source grounding mode, when the initial value of the voltage source is set as unbalanced, the theoretical output voltage of the voltage source is 4.345∠179.1° kV. The simulation results are shown in Fig. 10. Double closed-loop PI control can continuously stabilize the output voltage waveform of the controllable voltage source, and the adjusted amplitude can quickly approach the theoretical value of 4.345 kV. The maximum deviation is 0.16%, the phase can be stabilized at the theoretical 179.1°, the deviation is 0%, the amplitude stabilization time increases with the increase of the grounding transition resistance, Rf is 10 kΩ, when the residual voltage is less than 200 V, the amplitude and phase stabilization time of the PI controller is about 2.4 s. When the residual voltage is below 10 V, the amplitude and phase stabilization time of PI controller is about 5.5 s. Figure 10a shows the amplitude stabilization process, and Fig. 10b shows the phase stabilization process, with the maximum amplitude deviation of 7 V and phase deviation of 0° compared with the theoretical value.

**Figure 11:**
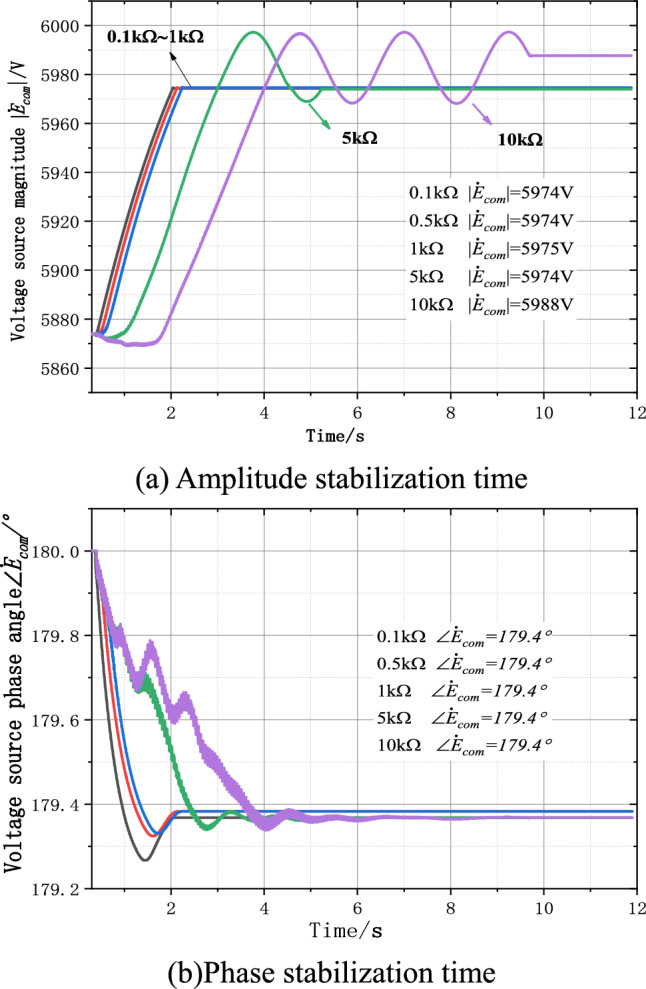
PI controller output and time relationship.

In the parallel arc suppression coil grounding mode, when the initial value of the controlled voltage source is set to be unbalanced, the theoretical output voltage of the voltage source is 5.982∠179.4° kV. The simulation result is shown in Fig. 11. Double closed-loop PI control can continuously stabilize the output voltage waveform of the controlled voltage source, and the adjusted amplitude can quickly approach the theoretical value of 5.87 4 kV. The maximum deviation is 1.9%, the phase can be stabilized at the theoretical 179.4°, the phase deviation is 0%, and the amplitude stabilization time increases with the increase of the grounding transition resistance. When the Rf is 10 kΩ and the residual voltage is below 200 V, the amplitude and phase stabilization time of the PI controller is about 4.1 s. When the residual voltage is less than 10 V, the amplitude and phase stabilization time of PI controller is about 9.1 s. The deviation between the output voltage amplitude and the phase stability of the voltage source and the theoretical value is larger than that of the independent controlled voltage source grounding method.

### Analysis of residual pressure elimination effect

The above analysis shows that the double closed-loop PI controller based on zero sequence voltage of neutral point can provide stable output voltage of controllable voltage source in distribution network under neutral point independent controllable voltage source grounding mode. The following will further discuss the relationship between residual voltage of fault phase and stability time under various imbalance degrees, as well as the magnitude of residual voltage and stability time under different transition resistance conditions.

**Figure 12:**
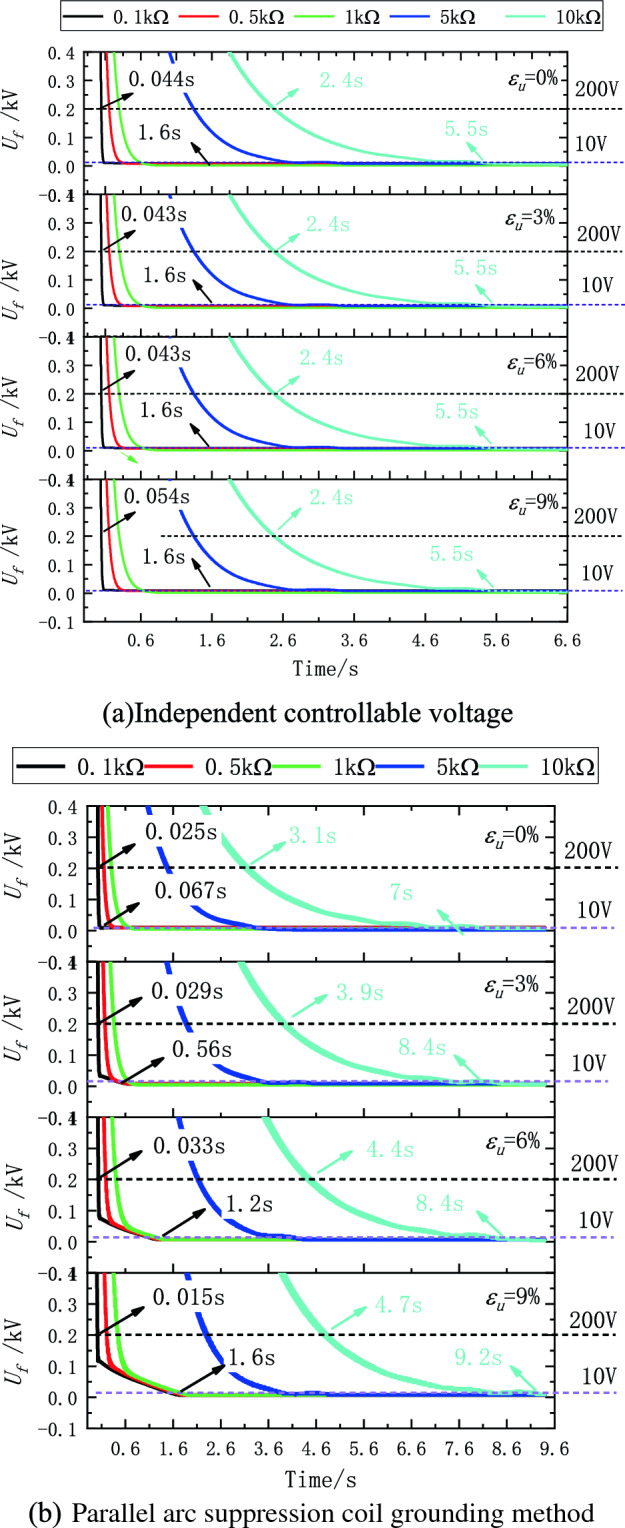
The relationship between unbalance degree, transition resistance, residual voltage and stability time is fully compensated.

Under the independent controllable voltage source grounding mode, the research shows that the unbalance degree of the same grounding transition resistance in Fig. 12a has no obvious correlation with the residual voltage stabilization time. When the transition resistance is 0.1kΩ, the increase of the unbalance degree has little influence on the residual voltage stabilization time, and the stabilization time of the residual voltage less than 200 V in the range of 0–9% of the unbalance degree $\varepsilon_{u}$ is about 44 ms. When the unbalance degree $\varepsilon_{u}$ is 0–9%, the residual voltage stabilization time increases with the increase of the transition resistance. When the fault resistance is 0.1–10 kΩ, the residual voltage stabilization time is 43 ms ~ 2.4 s at 200 V, and the stability time is 1.6–5.5 s at 10 V. When the ground transition resistance is 0.1–10kΩ under any working condition, the controlled voltage source can limit the fault residual voltage to less than 10 V and the fault residual current to less than 0.1A after stable output.

In the parallel arc suppression coil grounding mode, there is no obvious correlation between the unbalance degree of the same grounding transition resistance and the residual voltage stabilization time as shown in Fig. 12b. When the transition resistance is 0.1kΩ, the increase of the unbalance degree $\varepsilon_{u}$ has little influence on the residual voltage stabilization time. When the residual voltage is less than 200 V in the range of 0–9%, the stabilization time is about 15–33 ms. The stability time is close to that of the ground mode with independent controlled voltage source. When the unbalance $\varepsilon_{u}$ is 0–9%, the residual voltage stabilization time increases with the increase of the transition resistance. When the fault resistance is 0.1–10 kΩ, the residual voltage stabilization time is 43 ms to 4.7 s at 200 V, and the stability time is 67 ms to 9.2 s at 10 V. As shown in Fig. 12, when the ground transition resistance is 0.1–10kΩ under any working condition, the controlled voltage source can limit the fault phase residual voltage to less than 10 V and the fault residual current to less than 0.1A after stable output. For the 10 kV balanced distribution network, literature^27^ proposes a dual closed-loop control method consisting of a neutral point voltage outer loop and an output current inner loop, which can suppress the fault point voltage to 30 V. For the 10 kV unbalanced distribution network, the active arc-suppression device feeding back phase voltages to the neutral point proposed in literature^28^ can suppress the fault phase voltage to 311 V. A controllable voltage source control model proposed in literature^29^ can control the fault phase voltage to 40 V. The dual closed-loop control method proposed in this paper can suppress the fault phase voltage to 10 V, exhibiting good steady-state and dynamic performance.

### Control algorithm testing and validation

**Figure 13:**
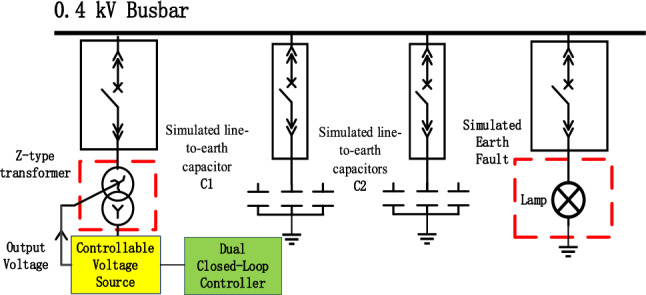
0.4 kV system earth fault simulation arc-extinguishing electrical wiring.

This section constructs a 0.4 kV distribution network test platform, as shown in Fig. 13. The figure consists of a 0.4 kV busbar, simulated line-to-earth capacitors C1 and C2, a simulated earth-fault lamp, an earth-fault transformer, a controllable voltage source, and a dual closed-loop controller. When the simulated earth fault lamp goes out, it indicates that the fault point arc is suppressed. The neutral point displacement voltage is 0 V or 25.5 V, grounded through 10Ω, 100Ω, and 500Ω transition resistors.

**Figure14:**
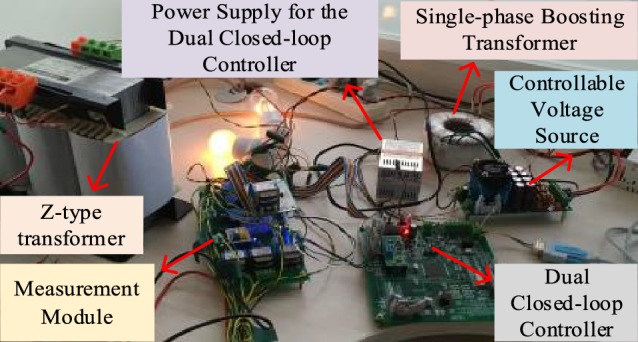
0.4 kV system earth fault arc-extinguishing test.

Figure 14 is a physical diagram of the simulation test, including a Z-type transformer, single-phase boosting transformer, controllable voltage source, dual closed-loop controller, measurement module, and power supply for the dual closed-loop controller.

The compensation effect of the controllable voltage source was tested under two working conditions, A phase grounded through resistances of 10Ω, 100Ω, and 500Ω, respectively. The time required for the controllable voltage source, under the control of the dual closed-loop controller, to suppress the voltage of the faulted phase (A phase) to below 2 V after its activation is shown in Table 3.

**Table 3:** Simulated experiment results.

Parameter	Value					
	Working condition 1		Working condition 2			
C/uF	C_A_ = 3.3uF C_B_ = 3.3uF C_C_ = 3.3uF		C_A_ = 3.3uF C_B_ = 4.5uF C_C_ = 2.1uF			
$\varepsilon_{u}$*/*%	0%		11%			
$\dot{E}_{0}$*/V*	0		25.5∠112°			
*L*/H	0.67H					
*R*_d_/Ω	10	100	500	10	100	500
$U_{f}$*/V*	2.0	1.4	1.1	1.9	1.3	1.1
Stabilization time/s	1.8	0.66	0.65	1.9	0.65	0.72

The dual closed-loop control algorithm proposed in this paper is validated for arc extinguishing performance in a 0.4 kV low-voltage system. Its effectiveness surpasses the calculated results of a 10 kV system simulated using PSCAD. However, there are significant limitations when comparing the 0.4 kV system to the 10 kV system, such as constraints on the transition resistance not being too large and the capacitance current being relatively small. The 0.4 kV control algorithm testing and verification platform serve as a reliable means for the project team to initially test the algorithm's reliability. It is only suitable for verifying the feasibility of the algorithm. Currently, the project team is developing a double-closed-loop control device for arc extinguishing in an unbalanced 10 kV distribution network with controllable voltage sources, aiming to further advance the practical application of the control algorithm.

## Conclusion

The problem addressed concerns the effectiveness of arc extinction in controllable voltage sources, influenced by the zero-sequence voltage resulting from unbalanced ground parameters in distribution networks. A dual closed-loop PI control method based on neutral point zero-sequence voltage is proposed, characterized by strong real-time control and minimal steady-state error, ensuring stable control of fault point current to zero and achieving reliable arc extinction. Feasibility is confirmed through simulation on a 10kV system and testing on a 0.4kV low-voltage platform, leading to the following conclusions:

1. Under the independent controllable voltage source grounding mode, the maximum residual pressure $\dot{U}_{f\_ignore}$ without considering the influence of imbalance is 124 V, and under the parallel arc suppression coil grounding mode, the maximum residual pressure $\dot{U}_{f\_ignore}$ without considering the influence of imbalance is 172 V. After the method proposed in this paper is adopted, the fault residual pressure $\dot{U}_{f\_mark}$ under stable conditions under each working condition is less than 10 V under the two grounding modes.
2. Under the independent controllable voltage source grounding mode, the PI controller's amplitude and phase stabilization time is about 2.4 s when the residual voltage is below 200 V, and the PI controller's amplitude and phase stabilization time is about 5.5 s when the residual voltage is below 10 V. And under the parallel arc suppression coil grounding mode, When the residual voltage is below 200 V, the amplitude and phase stabilization time of the PI controller is about 4.1 s, and when the residual voltage is below 10 V, the amplitude and phase stabilization time of the PI controller is about 9.1 s,The PI controller has good stability.
3. When the transition resistance is 0.1–10 kΩ, the time for the residual voltage to stabilize at 200 V is 43 ms ~ 2.4 s for the independent controlled voltage source grounding mode, 43 ms ~ 4.7 s for the parallel arc suppression coil grounding mode, and 1.6 s ~ 5.5 s for the stability at 10 V, The fastest stable time is 43 ms, and the dynamic performance is good.
4. When the residual voltage is below 200 V, the PI controller's amplitude and phase stabilization time are about 4.1 s; when the residual voltage is below 10 V, the PI controller's amplitude and phase stabilization time are about 9.1 s, and the residual current is less than 0.1 A, which provides a method for fully compensated arc elimination of controllable voltage source in unbalanced distribution network.

## Data Availability

The datasets used and/or analysed during the current study available from the corresponding author on reasonable request.
